# The Association of Oral Function with Oral Health-Related Quality of Life in University Students: A Cross-Sectional Pilot Study

**DOI:** 10.3390/ijerph17134863

**Published:** 2020-07-06

**Authors:** Saori Oku, Kiyomi Iyota, Shinsuke Mizutani, Shohei Otsuki, Kyohei Kubo, Shutaro Yamada, Yoshihiro Kobayashi, Haruhiko Kashiwazaki

**Affiliations:** 1 Section of Geriatric Dentistry and Perioperative Medicine in Dentistry, Division of Maxillofacial Diagnostic and Surgical Sciences, Faculty of Dental Science, Kyushu University, 3-1-1 Maidashi, Higashi-ku, Fukuoka 812-8582, Japan; saori.oku@dent.kyushu-u.ac.jp (S.O.); kibmc@sa2.gyao.ne.jp (K.I.); 3dd16015n@s.kyushu-u.ac.jp (Y.K.); kashi@dent.kyushu-u.ac.jp (H.K.); 2 OBT Research Center, Faculty of Dental Science, Kyushu University, 3-1-1 Maidashi, Higashi-ku, Fukuoka 812-8582, Japan; 3 Faculty of Dental Science, Kyushu University, 3-1-1 Maidashi, Higashi-ku, Fukuoka 812-8582, Japan; 1DD15011G@s.kyushu-u.ac.jp (S.O.); 1DD15017M@s.kyushu-u.ac.jp (K.K.); 1DD15051P@s.kyushu-u.ac.jp (S.Y.)

**Keywords:** oral function, quality of life, university students, cross-sectional study

## Abstract

The aim of this cross-sectional study was to investigate the association between oral function and oral health-related quality of life (OHRQoL) in healthy university students. Oral functions and OHRQoL (General Oral Health Assessment Index; GOHAI) were investigated in 58 healthy university students. Oral functions, such as tongue pressure, tongue-lip motor function, occlusal force, and masticatory function, were examined. The participants were divided into two groups based on low and high GOHAI scores. Information about oral health, dental caries treatment history, insomnia, and personality and lifestyle was obtained using a self-reported questionnaire. Oral mucosal wetness scores and tongue-lip motor functions (oral diadochokinesis /ka/) were significantly decreased in the low GOHAI score group compared to the high GOHAI score group (*p* = 0.001 and *p* = 0.017, respectively). In the logistic regression model, the GOHAI score was independently associated with the oral mucosal wetness score (odds ratio (OR) = 0.622; 95% confidence interval (CI), 0.411–0.941; *p* = 0.025) and oral diadochokinesis /ka/ (OR = 0.376; 95% CI, 0.170–0.832; *p* = 0.016). Our study demonstrated the presence of low oral function in university students and suggested its association to low OHRQoL in this population.

## 1. Introduction

Oral health-related quality of life (OHRQoL) represents the subjective experience of symptoms related to oral conditions that impact the well-being of an individual [1]. It is known to have important implications in the field of dentistry. For example, it can be used as an outcome measure to assess the determinants of oral health and to evaluate the effectiveness of dental treatments [2]. Therefore, it might be useful to investigate the factors related to OHRQoL in individuals across various generations and in patients with various diseases.

Previous studies on OHRQoL have shown its utility in diverse populations, including head and neck cancer patients [3], patients with tooth discoloration [4], and toddlers with early childhood caries [5]. Low OHRQoL has been associated with an increase in decayed teeth in children [6], xerostomia in younger adults [7], and malocclusion in university students [8]. On the other hand, a recent systematic review reported a negative association between OHRQoL and tooth loss or dental caries in adults [9]. Thus, the literature is inconsistent. Moreover, few studies have investigated the relationship between oral function, such as masticatory function, and OHRQoL in young adults.

Oral functions, such as speaking and swallowing, are extremely important for oral health. Saliva affects important aspects of life, such as the enjoyment and ingestion of food [10], whereas the tongue plays a role in holding the bolus within the oral cavity and transporting it to the pharynx. The base of the tongue generates the swallowing pressure within the pharynx. Hence, a previous study on improving oral functions not only in the elderly, but also in young adults, has been published [11]. This study reported the effectiveness of the strengthening of the tongue in young adults to prevent the development of low tongue pressure in the future.

In the present study, we hypothesized that the deterioration of oral function is associated with a decline in OHRQoL in university students. The aim of this cross-sectional study was to investigate the association between oral function and OHRQoL in healthy university students who do not have decayed teeth and acute gingival inflammation.

## 2. Materials and Methods

### 2.1. Study Population

Sixty-five participants (aged 20–29) were recruited by displayed posters written the aim and content of this research between November and December 2018 at the Kyushu University, Fukuoka, Japan. The participants were males and females who were not diagnosed with any systemic diseases and were not taking any medications. The exclusion criteria included the presence of decayed teeth, acute oral or general health-related symptoms (n = 0), receipt of dental treatment at the time of the study (n = 0), and receipt of previous orthodontic treatment (n = 7). Therefore, 58 university students were analyzed.

This study was approved by the Kyushu University Institutional Review Board for Clinical Research (Approval number: 30-314). All participants provided written informed consent before oral examination.

### 2.2. Sample Size Calculation

Since this was a pilot study, the sample size was not calculated. However, we collected 65 participants with reference to the previous study, which reported the relationship between xerostomia and OHRQoL (n = 62) [12].

### 2.3. Investigated Items

The participants underwent investigations comprising the “Oral Function Examination,” “OHRQoL,” and “Questionnaires,” as detailed below. These investigations were performed by two well-trained dentists with ≥6 years of experience on the same day. After training and calibration, the oral wetness score, tongue pressure, and occlusal force were recorded twice within a one-week interval in three volunteers by two dentists. The intra- and inter-examiner reliability was determined using the weighted Kappa statistic (≥0.60).

### 2.4. Oral Function Examination

The measurements of oral hypofunction advocated by the Japanese Society of Gerodontology in 2016 [13] were used in this study. Seven clinical conditions (oral hygiene, oral dryness, tongue pressure, tongue-lip motor function, occlusal force, masticatory function, and swallowing function) were measured.

#### 2.4.1. Oral Hygiene

The degree of tongue coating was assessed by visual inspection using the Tongue Coating Index (TCI) [14]. The tongue surface was divided into nine parts, and each was assessed from 0 to 2 points for degree of tongue coating: 0 indicated no coating on the tongue. The total score ranged 0–18 points. TCI was quantified as a percentage of the total score for each participant divided by the maximum score 18. The participant was diagnosed with poor oral hygiene when the TCI was more than 50% [13].

#### 2.4.2. Oral Wetness

An oral moisture checker (Mucus^®^, Life Co., Ltd., Saitama, Japan) was used to measure oral mucosal wetness at the center of the dorsum of the tongue, 2 h after a meal under non-stressful circumstances [15]. The center of the lingual mucosa, approximately 10 mm from the tip of the tongue, was pressed with a force of 200 g for 2 s for three trials. The median was assessed as the measured value. The participant was diagnosed with oral dryness if the measured value was less than 27.0 [13].

#### 2.4.3. Tongue Pressure

The maximum tongue pressure was measured using a tongue pressure measuring instrument (JMS tongue pressure device; JMS Co., Ltd., Hiroshima, Japan). The tongue pressure determined by the instrument is the reading exerted when the participant compresses a balloon attached to a tongue pressure probe onto the anterior palate for a few seconds using the maximum voluntary force of the tongue. Measurements were taken three times, and the participant was diagnosed with decreased tongue pressure when the maximum tongue pressure obtained was less than 30 kPa [13].

#### 2.4.4. Tongue-Lip Motor Function

Oral diadochokinesis comprises comprehensive measurements of the motor speed and dexterity of the tongue and lips. The participants produced each of the syllables /pa/, /ta/, and /ka/ repeatedly for 5 s. The number of respective syllables produced per second was determined using an automatic counter (Kenkokun Handy^®^, Takei Scientific Instruments Co., Ltd., Niigata, Japan) [16]. The participant was diagnosed with decreased tongue-lip motor function when the number of any of /pa/, /ta/, or /ka/ syllables produced was less than six per second [13].

#### 2.4.5. Occlusal Force

The occlusal force of the whole dentition was measured using a pressure indicating film (Dental Prescale II; GC Co., Tokyo, Japan) when the participants clenched their teeth for 3 s in the intercuspal position. The films were analyzed with an occluzer scanner (Bite Force Analyzer; GC Co., Tokyo, Japan), and the participant was diagnosed with reduced occlusal force when the measured value was less than 500 N [17].

#### 2.4.6. Masticatory Function

Masticatory function was assessed by the concentration of glucose obtained after chewing 2 g of gummy jelly. After chewing for 20 s, the participants rinsed their mouth with 10 mL of water for several seconds and spit into a cup with a filter mesh. The glucose concentration in the solution that passed through the mesh was then measured using a masticatory ability testing system (Gluco Sensor GS-II, GC Co., Tokyo, Japan) [18]. The participant was diagnosed with decreased masticatory function if the concentration of glucose was less than 100 mg/dL [13].

#### 2.4.7. Swallowing Function

Swallowing function was assessed by a self-administered questionnaire for swallowing screening (the 10-item Eating Assessment Tool (EAT-10)) [19]. The EAT-10 consists of 10 questions, each of which is answered on a 5-point scale from 0 (no problem) to 4 points (severe problem), and the total score ranges 0–40 points. A diagnosis of suspected dysphagia is reached when the total score is 3 or higher [13].

### 2.5. OHRQoL

The General Oral Health Assessment Index (GOHAI), a widely used tool in clinical and epidemiologic studies, was used to assess OHRQoL [20]. Although originally developed for the elderly, it has also been recently used to evaluate young adults [21,22]. The GOHAI consists of three subscales of oral health: Physical functions (eating, pronunciation, and swallowing), psychosocial impacts (aesthetics and social interaction), and pain and discomfort (sensitive teeth and use of medicine due to toothache). In the present study, the participants answered 12 questions regarding the frequency of oral health symptoms over the past three months. Each question is answered on a 5-point scale from 1 (never) to 5 (always), and the total score ranges 12–60 points. A higher score indicates lower OHRQoL.

### 2.6. Questionnaires

The participants completed a self-administered questionnaire before the oral examination. The questionnaire included sex, age, body mass index, and other items that are discussed below.

#### 2.6.1. Oral Health and Dental Caries Treatment History

The participants were asked the following questions regarding their oral health and their dental caries treatment history:

(1) Daily tooth brushing: How often do you brush your teeth daily? (three times or more, two times, one time or less)
(2) Usage of dental floss: Do you use dental floss or interdental brush? (Yes/No)
(3) Regular dental check-up: Have you had a regular check-up at a dental clinic in the past year? (Yes/No)
(4) Dental caries treatment history: Have you ever received dental caries treatment? (Yes/No)

Furthermore, an examiner confirmed by interview and examination that the participants did not have gingival pain, bleeding, or swelling.

#### 2.6.2. Athens Insomnia Scale

The Athens Insomnia Scale (AIS) is a short and widely used self-reporting tool for insomnia assessment. It consists of eight questions that are designed to estimate the participants’ sleep difficulties that have occurred at least three times per week during the past month. Each question is answered on a 4-point scale from 0 (no problem) to 3 (sleep difficulty) and the total score ranges from 0 to 24 points. A total score of 6 points or higher indicates pathological insomnia [23].

#### 2.6.3. Type D Personality

Type D personality is a personality that is a combination of “negative affectivity” and “social inhibition.” It was assessed using the 14-item Type D Personality Scale (DS14) [24], which consists of the two above-mentioned personality subscales, each with 7 questions. Each question is answered on a 5-point Likert-type scale from 0 (false) to 4 (true) and the total score ranges from 0 to 28. The participants with scores above 10 on both subscales were defined as Type D personality [24].

#### 2.6.4. Lifestyle

The factors assessed regarding lifestyle were as follows: Regular alcohol consumption (yes/no), smoking status (yes/no), and exercise habit (yes/no).

### 2.7. Statistical Analysis

The median of the GOHAI national norm for Japanese individuals aged 20–29 years was 55.0 [25]. Therefore, the participants were divided into two groups based on the GOHAI score: High score (≥56) and low score (≤55). Then, the Mann–Whitney *U* test or the Chi-square test was used to determine whether there were any significant differences between the two GOHAI groups. Using a logistic regression model, both odds ratios (ORs) and 95% confidence intervals (CIs) were calculated. The GOHAI groups were used as dependent variables. Independent variables were selected when the *p*-value was <0.20 on the Mann–Whitney *U* test or Chi-square test for each variable. Moreover, to examine the relationship between oral function and the three domains of the GOHAI, nonparametric correlation coefficients (Spearman’s (*p*)) were used.

*p* < 0.05 was considered significant. Analyses were performed using the SPSS software program (version 26.0, IBM Tokyo, Japan).

## 3. Results

Table 1 shows the characteristics of the 58 participants (33 males and 25 females, aged 20–29) in this study. The percentage of participants who had previous dental caries treatment history was 12%, and almost all participants brushed their teeth more than two times every day. About half of the participants had a Type D personality. The median of the GOHAI was 56, which was close to the national norm (55) for Japanese individuals aged 20–29. The most common oral hypofunction was oral dryness (24%), followed by decreased tongue-lip motor function (oral diadochokinesis /pa/ and /ka/). On the contrary, none of the participants demonstrated poor oral hygiene.

Table 2 shows the comparison of variables between the two groups based on the GOHAI scores. No significant differences in lifestyle habits, oral health behaviors, and personality were observed between the two groups. The mucus score in the low GOHAI score group was significantly lower than that in the high score group (*p* = 0.001; Table 3), indicating that an increased number of participants in the low score group had oral dryness. Tongue-lip motor function (oral diadochokinesis /ka/) was decreased in the low score group (*p* = 0.017) compared to the high score group. However, no differences in other tongue-lip motor functions (oral diadochokinesis /pa/ and /ta/) were noted between the two groups.

In the logistic regression model, a low GOHAI score was independently related to mucus score (OR = 0.622; 95% CI, 0.411–0.941; *p* = 0.025) and oral diadochokinesis /ka/ (OR = 0.376; 95% CI, 0.170–0.882; *p* = 0.016), following adjustment for confounding factors such as tongue pressure and masticatory function (Table 4). Thus, decreased oral wetness and decreased movement of the tongue tip worsens OHRQoL. The Nagelkerke R^2^ value of this model was 0.259. The accuracy of discrimination was 55.2%, and the Hosmer–Lemeshow test found the model fit to be acceptable, with a Chi-square statistic of 10.433 (*p* = 0.236).

The associations between oral function and the three subscales of GOHAI (physical functions, psychosocial impacts, and pain and discomfort) are shown in Table 5. Oral dryness was significantly associated with the three subscales (*p* < 0.05). Moreover, tongue-lip motor function (oral diadochokinesis /ka/) was significantly associated with physical functions and pain and discomfort, which means that participants who had better tongue-lip motor function (oral diadochokinesis /ka/) experienced better quality of life. On the contrary, a weak negative association between low oral function (tongue pressure and masticatory function) and high quality of life was observed (*p* < 0.05).

## 4. Discussion

The present study is the first to reveal the actual state of oral functions in university students and demonstrate that low oral function may be associated with low OHRQoL. Whereas oral hypofunction in older people has been the focus of attention in Japan [13], information about oral hypofunction in young adults is lacking. In the current study, we adopted the measurement methods and diagnostic criteria used for patients aged 65 or above. To the best of our knowledge, this is the first study to demonstrate oral hypofunction conditions in university students.

The most common oral hypofunction in this study was oral dryness (24%). Previous studies have reported that the prevalence of xerostomia is 9.9% in younger adults [7] and 8.8% in university students [26]. The discrepancy in the prevalence rate may be attributed to the different measurement methods used. The oral moisture checker was used to determine xerostomia in the current study, whereas the standard dry mouth question was used in the previous studies. It might be interesting to examine the differences in the scores obtained using moisture-checking devices and those obtained using the subjective question in young adults. Several participants were diagnosed with decreased tongue-lip motor function, which may be attributed to oral dryness, because saliva is known to play an important role in speech [27].

The logistic regression model indicated that the low OHRQoL (low GOHAI score) was independently associated with oral dryness and low tongue motor function (diadochokinesis /ka/). Whereas the association between oral dryness and OHRQoL in our study is consistent with that reported previously [7], there are no reports on the relationship between tongue motor function and OHRQoL. The diadochokinesis /ka/ is used to evaluate motor functions in the posterior region of the tongue [7]. Therefore, the association between diadochokinesis /ka/ and the physical functions domain in the GOHAI scale may be reasonable. On the contrary, the direct association between tongue motor function and pain and discomfort remains unclear. In addition to this association, further studies are required to investigate the negative association between low oral function (tongue pressure and masticatory function) and high quality of life.

Although a recent systematic review shows that individuals with a Type D personality possess a higher risk for consistently impaired health-related quality of life over time than those with non-Type D [28], the relationship between OHRQoL and Type D personality has not been demonstrated so far. In the current study, no statistically significant differences in GOHAI scores were observed between the Type D and non-Type D personalities. Thus, this finding is new knowledge, but long-term observation may be necessary, because the participants were younger and healthier than those in previous studies [29]. The relationship between insomnia and low GOHAI scores in elderly people has been reported in a previous study [30]. However, no such relationship was observed in the current study. This discrepancy may be related to the fact that some participants in the previous study used sleep medications, whereas none of the participants in the present study required any sleep medications, probably due to differences in age.

The previous study examined the oral clinical status using scores for decayed, missing, and filled teeth (DMFT) or periodontal status. Previous findings suggested that a high DMFT score was correlated with anxiety, which affects the self-rated oral health [31]. Furthermore, a high DMFT score is a primary factor for low OHRQoL in children [6]. Although one study showed no relationship between clinical periodontal conditions and OHRQoL in young adults [32], others have suggested correlations in patients with obvious symptoms of periodontitis [33]. To minimize these influences, we excluded the participants who had decayed teeth and acute gingival inflammation. However, the examiners checked only their decayed teeth and participants were only asked about their dental caries treatment history. The number of filled teeth was not confirmed and DMFT score and pocket probing depth were not examined. We should have considered the presence of metal dental prosthesis, which may have influenced the aesthetics.

The relationship between malocclusion and OHRQoL has been established in a previous study [8]. We excluded students with a history of previous orthodontic treatment but did not evaluate malocclusion because our examiner was not an orthodontic specialist. Future studies should evaluate malocclusion.

The short form of the Oral Health Impact Profile (OHIP-14) is considered to be a reliable and valid tool for assessing OHRQoL in the clinical setting, as is the GOHAI [34]. Whereas the OHIP-14 scale focuses on the impact of pain on psychological and behavioral traits, the GOHAI scale focuses on functional limitation related to pain [35]. Since some previous studies have assessed OHRQoL using both OHIP-14 and GOHAI [35,36], further study of the relationship between OHIP-14 and oral function is required.

There are some limitations to this study. First, it was a cross-sectional study, so causal associations could not be concluded. Second, other possible confounders related to GOHAI, such as health-related quality of life [30], education level [4], income [4], and temporomandibular joint disorder [37], were not evaluated. Furthermore, the diagnostic criteria used in this study were originally established for patients with age >65 years, so they might not be appropriate for university students. In addition, all participants were recruited from Kyushu University, which might limit the ability to extrapolate these findings to the general population of young adults. Finally, this was a pilot study and the sample size was small. Larger longitudinal studies are needed to explore the other factors and confirm the relationship between oral function and OHRQoL.

## 5. Conclusions

Our study showed the presence of low oral function and its possible association with low OHRQoL in healthy university students without decayed teeth or acute gingival inflammation. Therefore, it might be necessary to focus on not only conventional oral diseases, such as tooth decay, but also oral functions for the maintenance and improvement of oral health.

## Figures and Tables

**Table 1:** Characteristics of the study participants.

Variables			Total (n = 58)
Age (years)			22 (22, 24)
Sex		Male	33 (57)
Body mass index			20.0 (18.8, 22.2)
Daily tooth brushing		≥2 times	56 (97)
Regular dental check-up		Yes	22 (38)
Usage of dental floss		Yes	28 (48)
Dental caries treatment history		Yes	7 (12)
Number of natural teeth		<20	0 (0)
Smoking habit		Yes	3 (5.2)
Alcohol consumption		Yes	38 (67)
Exercise habit		Yes	33 (57)
Athens Insomnia Scale			3.5 (2.2, 5.0)
Type D personality		Yes	27 (47)
GOHAI			56 (53, 58)
Oral hypofunction			
Poor oral hygiene		≥50% (TCI)	0 (0)
Oral dryness		<27.0 (Mucus score)	14 (24)
Decreased tongue pressure		<30 kPa	6 (10)
Decreased tongue-lip motor function	ODK /pa/	<6.0 times/second	12 (21)
	ODK /ta/	<6.0 times/second	1 (2)
	ODK /ka/	<6.0 times/second	9 (16)
Reduced occlusal force		<500 N	3 (5)
Decreased masticatory function		<100 mg/dL	3 (5)
Deterioration of swallowing function		≥3 (EAT-10 score)	2 (3)

Values are reported as medians (25th percentile, 75th percentile) or numbers (percentage). GOHAI: General Oral Health Assessment Index, TCI: Tongue Coating Index, ODK: Oral diadochokinesis.

**Table 2:** Comparison of variables in the two groups based on the GOHAI score.

Variables		Low Score Group (n = 26)	High Score Group (n = 32)	*p*-Value
Age (years)		22 (21, 23)	22 (22, 24)	0.472 *
Sex	Male	14 (54)	19 (59)	0.876 ^†^
	Female	12 (46)	13 (41)	
Body mass index	<18.5	3 (12)	6 (19)	0.809 ^†^
	18.5–24.9	21 (81)	24 (75)	
	≥25.0	2 (7.7)	2 (6.3)	
Daily tooth brushing	≥2 times	26 (100)	30 (94)	0.497 ^†^
	<2 time	0 (0)	2 (6.3)	
Regular dental check-up	Yes	10 (39)	12 (38)	1.000 ^†^
	No	16 (62)	20 (63)	
Usage of dental floss	Yes	14 (54)	14 (44)	0.598 ^†^
	No	12 (46)	18 (56)	
Dental caries treatment history	Yes	5 (19)	2 (6.3)	0.225 ^†^
	No	21 (81)	30 (94)	
Smoking habit	Yes	0 (0)	3 (9)	0.245 ^†^
	No	26 (100)	29 (91)	
Alcohol consumption	Yes	17 (65)	21 (66)	1.000 ^†^
	No	9 (35)	11 (34)	
Exercise habit	Yes	17 (65)	16 (50)	0.292 ^†^
	No	9 (35)	16 (50)	
Athens Insomnia Scale		3.5 (2.0, 5.0)	3.5 (1.0, 5.8)	0.787 *
Type D personality	Yes	14 (54)	13 (41)	0.428 ^†^
	No	12 (46)	19 (59)	

Values are reported as medians (25th percentile, 75th percentile) or numbers (percentage). GOHAI: General Oral Health Assessment Index, ODK: Oral diadochokinesis. * Mann–Whitney *U* test, ^†^ Chi-square test.

**Table 3:** Comparison of oral function in the two groups based on the GOHAI score.

Variables		Low Score Group (n = 26)	High Score Group (n = 32)	*p*-Value *
Oral hygiene (Tongue Coating Index)		0 (0, 0)	0 (0, 0)	0.522
Oral dryness (Mucus score)		27.4 (26.4, 28.1)	28.3 (27.7, 29.9)	0.001
Tongue pressure (kPa)		42.7 (40.7, 47.4)	39.2 (33.8, 45.7)	0.056
Tongue-lip motor function (times/second)	ODK /pa/	6.5 (6.0, 7.0)	6.6 (6.1, 7.0)	0.495
	ODK /ta/	7.2 (6.4, 7.6)	7.3 (6.7, 7.8)	0.433
	ODK /ka/	6.4 (6.0, 7.0)	7.0 (6.4, 7.6)	0.017
Number of natural teeth		28.0 (28.0, 28.0)	28.0 (28.0, 28.0)	0.966
Occlusal force (N)		1172 (686, 1442)	1079 (839, 1318)	0.704
Masticatory function (mg/dL)		170 (140, 215)	150 (124, 182)	0.103
Swallowing function (EAT-10 score)		0 (0, 0)	0 (0, 0)	0.611

Values are reported as medians (25th percentile, 75th percentile). GOHAI: General Oral Health Assessment Index, ODK: Oral diadochokinesis. * Mann–Whitney *U* test.

**Table 4:** Adjusted odds ratios and 95% CIs for low GOHAI score.

Variables		Odds Ratio	95% CI	*p*-Value
Oral dryness	Mucus score	0.622	0.411–0.941	0.025
Tongue-lip motor function (times/second)	ODK /ka/	0.376	0.170–0.832	0.016

Independent variable: Oral dryness (continuous), tongue-lip motor function/ka/ (continuous), tongue pressure (continuous), masticatory function (continuous). GOHAI: General Oral Health Assessment Index, ODK: Oral diadochokinesis.

**Table 5:** Association between oral functions and GOHAI score.

Variables		Subscales		
		Physical Functions	Psychosocial Impacts	Pain and Discomfort
Tongue Coating Index		−0.041	0.031	0.071
Oral dryness		**0.268**	**0.374**	**0.354**
Tongue pressure		−0.112	−0.203	**−0.268**
Tongue-lip motor function	ODK /pa/	0.143	0.017	0.049
	ODK /ta/	0.079	−0.053	0.042
	ODK /ka/	**0.286**	0.187	**0.262**
Number of natural teeth		0.171	0.039	−0.103
Occlusal force		−0.066	−0.148	−0.168
Masticatory function		−0.226	**−0.280**	**−0.283**
Swallowing function		0.041	0.001	−0.113

Values are reported as Spearman’s correlation coefficient. GOHAI: General Oral Health Assessment Index, ODK: Oral diadochokinesis. Bolds are significant associations (*p* < 0.05).

## References

[1] Locker D., Allen F. (2007). What do measures of ‘oral health-related quality of life’ measure?. Commun. Dent. Oral Epidemiol..

[2] Sischo L., Broder H.L. (2011). Oral Health-related quality of life: What, why, how, and future implications. J. Dent. Res..

[3] Schweyen R., Kuhnt T., Wienke A., Eckert A., Hey J. (2017). The impact of oral rehabilitation on oral health-related quality of life in patients receiving radiotherapy for the treatment of head and neck cancer. Clin. Oral Investig..

[4] Palacios R.D., Ramírez-Amador V., Jarillo-Soto E.C., Irigoyen-Camacho M.E., Mendoza-Núñez V.M. (2015). Relationship between gender, income and education and self-perceived oral health among elderly Mexicans. An exploratory study. Cien Saude Colet.

[5] Filstrup S.L., Briskie D., da Fonseca M., Lawrence L., Wandera A., Inglehart M.R. (2003). Early childhood caries and quality of life: Child and parent perspectives. Pediatr. Dent..

[6] Alsumait A., ElSalhy M., Raine K., Cor K., Gokiert R., Al-Mutawa S., Amin M. (2015). Impact of dental health on children’s oral health-related quality of life: A cross-sectional study. Health Qual. Life Outcomes.

[7] Thomson W.M., Lawrence H.P., Broadbent J.M., Poulton R. (2006). The impact of xerostomia on Oral Health-related quality of life among younger adults. Health Qual. Life Outcomes.

[8] Yamanae-Takeuchi M., Ekuni D., Mizutani S., Kataoka K., Taniguchi-Tabata A., Azuma T., Furuta M., Tomofuji T., Iwasaki Y., Morita M. (2016). Associations among oral health-related quality of life, subjective symptoms, clinical status, and self-rated oral health in Japanese university students: A crosssectional study. BMC Oral Health.

[9] Haag D.G., Peres K.G., Balasubramanian M., Brennan D.S. (2017). Oral conditions and health-related quality of life: A systematic review. J. Dent. Res..

[10] Cassolato S.F., Turnbull R.S. (2003). Xerostomia: Clinical aspects and treatment. Gerodontology.

[11] Yano J., Yamamoto-Shimizu S., Yokoyama T., Kumakura I., Hanayama K., Tsubahara A. (2019). Effects of tongue-strengthening exercise on the geniohyoid muscle in young healthy adults. Dysphagia.

[12] Herrmann G., Müller K., Behr M., Hahnel S. (2017). Xerostomia and its impact on oral health-related quality of life. Z. Gerontol. Geriatr..

[13] Minakuchi S., Tsuga K., Ikebe K., Ueda T., Tamura F., Nagao K., Furuya J., Matsuo K., Yamamoto K., Kanazawa M. (2018). Oral hypofunction in the older population: Position paper of the Japanese Society of Gerodontology in 2016. Gerodontology.

[14] Shimizu T., Ueda T., Sakurai K. (2007). New method for evaluation of tongue-coating status. J. Oral Rehabil..

[15] Murakami M., Nishi Y., Kamashita Y., Nagaoka E. (2009). Relationship between medical treatment and oral dryness diagnosed by oral moisture-checking device in patients with maxillofacial prostheses. J. Prosthodont. Res..

[16] Yamada A., Kanazawa M., Komagamine Y., Minakuchi S. (2015). Association between tongue and lip functions and masticatory performance in young dentate adults. J. Oral Rehabil..

[17] Ueda T., Minakuchi S., Tsuga K., Ikebe K., Tamura F., Nagao K., Furuya J., Matsuo K., Yamamoto K., Kanazawa M. (2018). Evaluation and diagnostic criteria for oral hypofunction-interim report for prospective revision. Jpn. J. Gerodontol..

[18] Uesugi H., Shiga H. (2017). Relationship between masticatory performance using a gummy jelly and masticatory movement. J. Prosthodont. Res..

[19] Belafsky P.C., Mouadeb D.A., Rees C.J., Pryor J.C., Postma G.N., Allen J., Leonard R.J. (2008). Validity and reliability of the Eating Assessment Tool (EAT-10). Ann. Otol. Rhinol. Laryngol..

[20] Naito M., Suzukamo Y., Nakayama T., Hamajima N., Fukuhara S. (2006). Linguistic adaptation and validation of the General Oral Health Assessment Index (GOHAI) in an elderly Japanese population. J. Public Health Dent..

[21] Atchison K.A., Der-Martirosian C., Gift H.C. (1998). Components of self-reported oral health and general health in racial and ethnic groups. J. Public Health Dent..

[22] Tubert-Jeannin S., Riordan P.J., Morel-Papernot A., Porcheray S., Saby-Collet S. (2003). Validation of an oral health quality of life index (GOHAI) in France Community. Commun. Dent. Oral Epidemiol..

[23] Soldatos C.R., Dikeos D.G., Paparrigopoulos T.J. (2003). The diagnostic validity of the Athens insomnia scale. J. Psychosom. Res..

[24] Denollet J. (2005). DS14: Standard assessment of negative affectivity, social inhibition, and Type D personality. Psychosom. Med..

[25] GOHAI. https://www.sf-36.jp/qol/files/gohai_norm.pdf..

[26] Mizutani S., Ekuni D., Tomofuji T., Azuma T., Kataoka K., Yamane M., Iwasaki Y., Morita M. (2015). Relationship between xerostomia and gingival condition in young adults. J. Periodont. Res..

[27] Dawes C., Pedersen A.M., Villa A., Ekström J., Proctor G.B., Vissink A., Aframian D., McGowan R., Aliko A., Narayana N. (2015). The functions of human saliva: A review sponsored by the World Workshop on Oral Medicine VI. Arch. Oral Biol..

[28] Huang I.C., Lee J.L., Ketheeswaran P., Jones C.M., Revicki D.A., Wu A.W. (2017). Does personality affect health-related quality of life? A systematic review. PLoS ONE.

[29] Pedersen S.S., Holkamp P.G., Caliskan K., van Domburg R.T., Erdman R.A., Balk A.H. (2006). Type D personality is associated with impaired health-related quality of life 7 years following heart transplantation. J. Psychosom. Res..

[30] Noguchi S., Makino M., Haresaku S., Shimada K., Naito T. (2017). Insomnia and depression impair oral health-related quality of life in the old-old. Geriatr. Gerontol. Int..

[31] Kojima A., Ekuni D., Mizutani S., Furuta M., Irie K., Azuma T., Tomofuji T., Iwasaki Y., Morita M. (2013). Relationships between self-rated oral health, subjective symptoms, oral health behavior and clinical conditions in Japanese university students: A cross-sectional survey at Okayama University. BMC Oral Health.

[32] Saho H., Ekuni D., Kataoka K., Taniguchi-Tabata A., Toyama N., Sugiura Y., Islam M.M., Iwasaki Y., Morita M. (2019). Structural equation modeling to detect predictors of oral health-related quality of life among Japanese university students: A prospective cohort study. Qual. Life Res..

[33] Eltas A., Uslu M.O., Eltas S.D. (2016). Association of Oral Health-related quality of life with periodontal status and treatment needs. Oral Health Prev. Dent..

[34] Locker D., Matear D., Stephens M., Lawrence H., Payne B. (2001). Comparison of the GOHAI and OHIP-14 as measures of the oral health-related quality of life of the elderly. Commun. Dent. Oral Epidemiol..

[35] Adamo D., Pecoraro G., Fortuna G., Amato M., Marenzi G., Aria M., Mignogna M.D. (2020). Assessment of oral health-related quality of life, measured by OHIP-14 and GOHAI, and psychological profiling in burning mouth syndrome: A case-control clinical study. J. Oral Rehabil..

[36] Rodakowska E., Mierzyńska K., Bagińska J., Jamiołkowski J. (2014). Quality of life measured by OHIP-14 and GOHAI in elderly people from Bialystok, north-east Poland. BMC Oral Health.

[37] Naito M., Yuasa H., Nomura Y., Nakayama T., Hamajima N., Hanada N. (2006). Oral Health status and health-related quality of life: A systematic review. J. Oral Sci..

